# Investigation of Liver X Receptor Gene Variants and Oxysterol Dysregulation in Autism Spectrum Disorder †

**DOI:** 10.3390/children11050551

**Published:** 2024-05-05

**Authors:** Tuğba Menteşe Babayiğit, Güvem Gümüş-Akay, Merve Çikili Uytun, Özlem Doğan, Muhittin A. Serdar, Gökçe Yağmur Efendi, Ayşe Gökçe Erman, Esra Yürümez, Didem Behice Öztop

**Affiliations:** 1 Department of Child and Adolescent Psychiatry, Aksaray University School of Medicine Training and Research Hospital, Aksaray 68100, Turkey; 2 Department of Physiology, Ankara University School of Medicine, Ankara 06100, Turkey; ggumusakay@ankara.edu.tr; 3 Brain Research Center (AUBAUM), Ankara University, Ankara 06340, Turkey; 4 Neuroscience and Neurotechnology Center of Excellence (NÖROM), Ankara University, Ankara 06560, Turkey; 5 Department of Child and Adolescent Psychiatry, Ankara University School of Medicine, Ankara 06100, Turkey; uytun@ankara.edu.tr (M.Ç.U.); eyurumez@ankara.edu.tr (E.Y.); oztop@ankara.edu.tr (D.B.Ö.); 6 Department of Biochemistry, Ankara University School of Medicine, Ankara 06100, Turkey; ozlemdogan@ankara.edu.tr; 7 Department of Medical Biochemistry, Acıbadem University School of Medicine, Ankara 06460, Turkey; muhittin.serdar@acibadem.edu.tr; 8 Department of Child and Adolescent Psychiatry, Kocaeli University School of Medicine, Kocaeli 41001, Turkey; yagmur.efendi@kocaeli.edu.tr; 9 Department of Basic Biotechnology, Ankara University Institute of Biotechnology, Ankara 06135, Turkey; agerman@ankara.edu.tr

**Keywords:** autism spectrum disorder, cholesterol, *NR1H2*, oxysterol, liver X receptor, single nucleotide polymorphism

## Abstract

The *NR1H2* gene produces the Liver X Receptor Beta (LXRB) protein, which is crucial for brain cholesterol metabolism and neuronal development. However, its involvement in autism spectrum disorder (ASD) remains largely unexplored, aside from animal studies. This study is the first to explore the potential link between autism and rs2695121/rs17373080 single nucleotide polymorphisms (SNPs) in the regulatory regions of *NR1H2*, known for their association with neuropsychiatric functions. Additionally, we assessed levels of oxysterols (24-Hydroxycholesterol, 25-Hydroxycholesterol, 27-Hydroxycholesterol), crucial ligands of LXR, and lipid profiles. Our cohort comprised 107 children with ASD and 103 healthy children aged 2–18 years. Clinical assessment tools included the Childhood Autism Rating Scale, Autistic Behavior Checklist, and Repetitive Behavior Scale-Revised. Genotyping for SNPs was conducted using PCR-RFLP. Lipid profiles were analyzed with Beckman Coulter kits, while oxysterol levels were determined through liquid chromatography–tandem mass spectrometry. Significantly higher total cholesterol (*p* = 0.003), LDL (*p* = 0.008), and triglyceride (*p* < 0.001) levels were observed in the ASD group. 27-Hydroxycholesterol levels were markedly lower in the ASD group (*p* ≤ 0.001). ROC analysis indicated the potential of 27-Hydroxycholesterol to discriminate ASD diagnosis. The SNP genotype and allele frequencies were similar in both groups *(p* > 0.05). Our findings suggest that disturbances in oxysterol metabolism, previously linked to neurodegeneration, may constitute a risk factor for ASD and contribute to its heterogeneous phenotype.

## 1. Introduction

According to the statistics provided by the Centers for Disease Control and Prevention (CDC) in 2023, the prevalence of autism spectrum disorder (ASD) is estimated to be 1 in 36 [1]. Over the past 30 years, there has been a significant rise in the number of individuals diagnosed with ASD, with rates increasing from 4.4/10,000 to currently reported levels. This increase has prompted researchers to intensify their efforts to uncover the underlying causes of the disorder [2].

The broad and heterogeneous phenotype characteristics of individuals with ASD indicate multiple risk factors interacting with each other rather than a single cause in the etiology of autism [3]. A hypothesis based on studies conducted in this field is that ASD is a “whole body disorder” that includes metabolic pathways expressed throughout the body and can cause different pathophysiological and clinical outcomes by interacting with other risk factors [4]. Several studies based on this hypothesis have reported that disruptions in cholesterol metabolism, which have an essential role in neuronal development, are observed in ASD and are associated with the development and severity of the disease [5]. Disturbances in cholesterol homeostasis can cause synaptic dysfunction by affecting neural development and synaptogenesis and thus lead to neuronal system disorders [6]. In addition, genetic syndromes characterized by disruptions in cholesterol homeostasis, such as Rett and Smith–Lemli–Opitz syndromes, have also been found to be highly associated with ASD [7].

In recent years, findings on cholesterol metabolism disturbances seen in ASD have been increasing. The *LXR*, which plays a central role in cholesterol metabolism, has become an important research focus because it has been shown to play an essential role in various neurodegenerative diseases such as Alzheimer’s, Parkinson’s, and multiple sclerosis (MS). These diseases share similarities with ASD including synaptic dysfunction, neuroinflammation, and neurodegeneration [8]. *LXR* belongs to the nuclear receptor superfamily of ligand-activated transcription factors and has two subtypes, *LXRα* (Encoder gene: *NR1H3*) and *LXRƁ* (encoder gene: *NR1H2*). *LXRα* is widely expressed in metabolically active tissues such as the liver, intestine, kidney, macrophages, and adipose tissue, whereas *LXRƁ* has a broader expression and is mainly expressed at high levels in developing brain tissue [9]. Oxysterols, the most important ligand of *LXR*, are formed in the cell in an amount proportional to the cholesterol content. Activated by the accumulation of oxysterol, *LXR*s function as cholesterol sensors that protect cells from cholesterol overload by activating the transcription of genes that reduce cholesterol synthesis and increase its excretion and degradation [10].

Research has shown the importance of *LXR* in neuronal processes, and increasing studies are exploring the connection between *LXR* and neuropsychiatric diseases. However, only a limited number of animal studies have investigated the correlation between ASD and *LXR*, and no clinical studies have yet been conducted [11,12]. Findings from animal modeling studies, which provide increasing evidence of the association between the *LXRƁ* mutation and autistic symptoms, suggest that LXR agonists may be a therapeutic target in this disorder. In an autism animal model, postnatal treatment with TO901317, an *LXR* agonist, activated neurogenesis-related genes in the hippocampus and reduced social deficits and repetitive behaviors through *LXRƁ*. Thus, it has been suggested that the *LXR* agonist is a potential treatment target for ASD [13].

Increasing evidence of cholesterol metabolism disruptions and *LXR* in neuropsychiatric diseases has also made *LXR* ligands, oxysterols, a research focus. Oxysterols play a role as endogenous regulators in a range of biological processes [14]. Oxysterols, found in very low concentrations in mammalian tissues, can reach high levels in many pathological conditions. Therefore, it is thought that these molecules formed in the excretion pathways of cholesterol might be used as biomarkers [15]. Changes in oxysterol levels have been reported in many neuropsychiatric disorders similar to ASD in terms of synaptic dysfunction [16]. To the best of our knowledge, only one study in the literature has examined the relationship between oxysterol levels and ASD. The study suggested that oxysterol, specifically 24-hydroxycholesterol (24-HC), might be a risk factor for the disorder and can serve as a diagnostic tool [17]. 24-HC is the most crucial oxysterol for brain cholesterol homeostasis and is also the most essential ligand of *LXRƁ*. 24-HC may contribute to functional changes in the brain through cytotoxicity, oxidative stress, apoptosis, and synaptic dysfunction, which are known to play a role in the pathogenesis of ASD [5].

Another oxysterol associated with neurodegenerative processes is 27-hydroxycholesterol (27-HC), which is the most crucial intermediate of peripheral cholesterol metabolism. Peripherally produced 27-HC can reach the brain by crossing the blood-brain barrier [18]. Numerous studies have shown alterations in the blood level of 27-HC in various neurodegenerative illnesses. In addition, some studies emphasize the importance of the balance between 24-HC and 27-HC for neuronal functions [19]. 25-Hydroxycholesterol (25-HC) is another oxysterol involved in peripheral cholesterol metabolism and is very similar to 24-HC and 27-HC in terms of molecular structure. To accurately measure the levels of other oxysterols, it must be eluted [20].

Our objective was to assess the lipid profiles (Total Cholesterol, Triglyceride, LDL, and HDL) as well as the levels of *LXR*’s significant ligands, 24-HC, 27-HC, and 25-HC, in children with ASD. We also compared these profiles with those of the non–autistic group. The gene encoding *LXRβ* (*NR1H2*), which is highly expressed in the developing brain and whose possible pathophysiological role in ASD has been demonstrated in animal studies, has also been indicated as a therapeutic target and has been previously studied in neuropsychiatric diseases [21]. Two SNPs, namely rs2695121 and rs17373080, located at the regulatory regions of the gene, which are thought to affect the expression and function of the genes, were investigated in both groups. To our knowledge, this is the first clinical study investigating the relationship between *LXR* and ASD. Our study also aimed to fill the gap in the literature by evaluating the lipid panel, the levels of 24-HC, 25-HC, and 27-HC, as well as clinical parameters in children with ASD using a large sample and wide age range and thus obtaining findings on whether the change pattern expected to be seen with age is different in children with ASD compared with healthy children. Therefore, the hypothesis that “oxysterols may be a biomarker in ASD” was also investigated, and it was aimed to evaluate the age and clinical situation in which this relationship may be guiding if any potential relationship is found.

## 2. Materials and Methods

### 2.1. Participants

After obtaining ethical approval, 107 children diagnosed with ASD and without any additional psychiatric diagnosis other than developmental delay/intellectual disability, aged 2–18, were included in the ASD group. Additionally, 103 children without any psychiatric diagnosis, matched with the ASD group in terms of age were included as the control group. Informed consent was obtained from both the children themselves and their parents; however, for those whose age and/or cognitive status were not at a sufficient level, consent was obtained solely from their parents. Exclusion criteria for both groups included being on any psychotropic medication, having any medical chronic disease, and/or parents having mental or physical problems that would prevent them from participating in the survey interviews.

### 2.2. Procedure

The children included in the study initially underwent a diagnostic psychiatric evaluation based on DSM-5 criteria, utilizing the Affective Disorders and Schizophrenia Form for School Age Children-Present and Lifelong Form DSM-5-Turkish Adaptation. Additionally, the Childhood Autism Rating Scale was completed for the ASD group. Following the diagnostic assessment, a sociodemographic characteristic form was completed to obtain a comprehensive medical and demographic history, and height–weight measurements were made. Subsequently, parents were asked to complete the Autism Behaviour Checklist and the Repetitive Behaviour Scale—Revised for the ASD group. Furthermore, all children diagnosed with ASD underwent a standardized developmental assessment or intelligence test appropriate for their developmental stage. After completing the psychiatric evaluations, peripheral blood samples were collected in the morning after an 8-h fasting period in both groups for biochemical and genetic analyses.

### 2.3. Clinical Assessment Tools

#### 2.3.1. Sociodemographic Data Form

The form filled out by the researcher during the interview with the parents included sociodemographic characteristics and information about the mother’s pregnancy history, child’s birth history, developmental stages, nutritional habits, infection history, dietary habits, and history of ASD diagnosis.

The Schedule for Affective Disorders and Schizophrenia for School-Age Children-Present and Lifetime Version, DSM-5 -Turkish Adaptation (K-SADS-PL-DSM-5-T).

This is a semi-structured diagnostic interview developed by Kaufman et al. to determine the past and current psychopathology of children and adolescents [22]. The Turkish translation, validity and reliability study of the form adapted to DSM-5 was conducted by Ünal et al. [23].

#### 2.3.2. Childhood Autism Rating Scale (CARS)

CARS, developed by Schopler et al. in 1971, is a behavioral rating scale that offers an objective and measurable assessment widely used in the diagnosis of autism and in differentiating children with autism from children with other developmental disorders [24]. Gassaloğlu, Baykara et al. (2016) conducted the validity and reliability analysis of the scale, and the cut-off score for autism diagnosis was reported as 29.5 [25]. In the original study, Cronbach’s alpha was 0.94, while in the Turkish version we used, it was determined as 0.95.

#### 2.3.3. The Autism Behaviour Checklist (ABC)

The scale developed by Krug et al. [26] assesses autism symptom areas, symptom severity, and clinical progression in school-age children with severe complaints. Yılmaz-Irmak et al. (2007) conducted a validity and reliability study of the Turkish version of the scale, and the cut-off score for the Turkish version of the scale was found to be 39 [27]. Regarding the scale’s reliability, Krug et al. (1993) found that the split-half reliability was 0.94. In the Turkish version, however, the Cronbach’s alpha coefficient was 0.92. The Turkish version of the scale was used in this study.

#### 2.3.4. The Repetitive Behaviour Scale—Revised (RBS-R)

The scale was developed by Bodfish et al. to evaluate repetitive behaviors and their severity [28]. As the score increases, the severity of repetitive behaviors is considered to increase. A validity and reliability study of the Turkish version of the scale was conducted by Akçamuş et al. in 2019 [29]. The Cronbach’s alpha coefficients for the subscales and total scores of the original version of the scale ranged from 0.71 to 0.90, while the Cronbach’s alpha coefficients for the Turkish version used in our study ranged from 0.73 to 0.94.

#### 2.3.5. Lipid Panel and Oxysterol Level Analysis

The lipid panel (Total cholesterol, Triglycerides, LDL, HDL) was studied on a Beckman Coulter 5800 (Beckmann Coulter, Inc. Brea. CA.US) AutoAnalyzer. The serum sample remaining after the lipid panel analysis was divided into at least two Eppendorfs and stored in an ultra-deep freezer at −80 °C until oxysterol analysis. Oxysterol analysis was performed by liquid chromatography-tandem mass spectrometry (LC-MS/MS) and Thermo Access Max Tandem Mass Spectrometer (San Jose, CA, USA) by modifying the method used by Sugimoto et al. [30]. The samples to be analyzed were added 10 µL PBS buffer, 2 mL sodium hydroxide, and 5 mL methanol and kept in a water bath at 50 °C for 1 h for saponification. The saponified samples were then treated with hexane, followed by extraction, and prepared for analysis. Calibrator, control, and internal standards were prepared by adding acetonitrile. The prepared calibrator, control, and samples were analyzed by LC-MS/MS, and chromatographic separation of 24-HC, 25-HC, and 27-HC hormones was performed. After detecting the separated hormones in the mass spectrometer, the quantification process of 24-HC, 25-HC, and 27-HC was initiated.

#### 2.3.6. Analysis of SNPs Identified for Liver X Receptor Beta

SNP analysis was performed using the PCR-RFLP (Polymerase CHain Reaction—Restriction Fragment Length Polymorphism) method. DNA samples were obtained from Ankara University Research Center Biobank. PCR-RFLP was performed using the Techne TC-412 Thermocycler device, and agarose gel electrophoresis was performed using Thermo EC320 and Mini Cell Primo. Each PCR employed a final concentration of 100 ng DNA, 10 pmol primer pairs, 1XPCR buffer containing KCl, 3 mM MgCl2, 200 μM each dNTP, and 1 U Taq DNA polymerase. The steps used for PCR thermal cycling were as follows: Initial denaturation at 94 °C for 5 min; 35 cycles of amplification at 94 °C for 30 s, 60 °C for 30 s, 72 °C for 30 s; and final extension at 72 °C for 7 min. The amplicons obtained were then digested with the Hpy188III enzyme, and the digestion products were separated and visualized by 3.5% agarose gel electrophoresis (Appendix A/Table A1).

### 2.4. Statistical Analysis

Statistical analyzes were performed using IBM SPSS Statistics for Windows 22.0. α = 0.05 was determined as the limit of significance. Descriptive statistics were given as mean ± standard deviation or median (minimum-maximum) for continuous variables depending on the fit of the data to normal distribution and frequency (percentage) for categorical variables. Chi-square or Fisher’s exact test was employed for the analysis of categorical data, while Student’s *t*-test/Mann–Whitney U test or one-way analysis of variance (ANOVA)/Kruskal–Wallis tests were used for the analysis of continuous data depending on the number of groups and whether the test normality assumptions were satisfied. Pearson or Spearman’s correlation coefficient was used to examine the co-variance of continuous variables, depending on whether the assumption of normality was satisfied or not. Receiver operating characteristic (ROC) curve analysis was performed to evaluate the diagnostic power of the oxysterol level.

## 3. Results

### 3.1. Data on Sociodemographic Characteristics

The sociodemographic characteristics of the groups are presented in Table 1. The groups had similar characteristics regarding family type, average age of parents, and education levels. While 22.4% (*n* = 24) of the ASD group consisted of girls and 77.6% (*n* = 83) of boys, 46.6% (*n* = 48) of the non–autistic group were girls and 53.4% (*n* = 83) were boys (*p* < 0.001).

### 3.2. Clinical Characteristics and Evaluation of Scales of the ASD Group

The clinical characteristics and ABC, CARS, and RBS-R scale scores of the ASD group are given in Table 2. In addition to special education, 26.2% (*n* = 28) of the children with ASD had at least one alternative treatment trial initiated by their families. 11 children, comprising 10.3% of the group, have been adhering to various dietary restrictions.

### 3.3. Lipid Profile Analysis

The lipid profile analysis of the groups is given in Table 3. The levels of total cholesterol, triglyceride, and LDL in the ASD group were significantly higher than those in the non–autistic group (*p* < 0.05), but there was no statistically significant difference in HDL levels (*p* > 0.05). There was no statistically significant difference between lipid levels and age in both groups. There was no statistically significant relationship between autism severity levels, developmental delay/intellectual disability status, clinical evaluation scale scores, and lipid levels of the children in the ASD group (*p* > 0.05). In addition, the lipid levels of the group on a restricted diet were similar to those of the group that did not follow a restricted diet (*p* > 0.05).

### 3.4. Oxysterol Analyzes

Oxysterol levels and comparisons between the groups are provided in Table 4. While there was no statistically significant difference between the groups regarding 25-HC, 24-S-HC, and 24-HC levels, 27-R-HC and 27-S-HC levels were significantly lower in the ASD group than in the non–autistic group. An examination of the correlation between oxysterol levels and age revealed that all oxysterols had a significant negative correlation with age in the non–autistic group. Levels of 25-HC, 24-S-HC, and 24-HC significantly negatively correlated with age in the ASD group.

**Figure 1:**
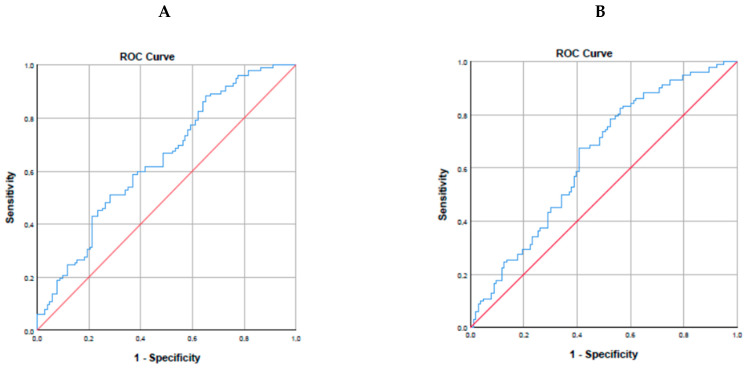
ROC curves for 27-S-HC (**A**) and 27-R-HC (**B**). (**A**): 27-S-HC: Cut-off value; 58.35 µg/L, the area under the curve; 0.644 (95% CI 0.569–0.719) sensitivity; 88% and specificity; 35%. (**B**): 27-R-HC: Cut-off value; 36.59 µg/L, the area under the curve; 0.639 (95% CI 0.563–0.715), sensitivity; 67%, and the specificity; 59%.

There was no statistically significant relationship between autism severity and oxysterol levels in the ASD group. In addition, the oxysterol levels of the group on a restricted diet were similar to those of the group that did not follow a restricted diet. In the ROC analysis performed to evaluate the predictive power of 27-S-HC level for autism, when the cut-off value was taken as 58.35 µg/L, the area under the curve was found to be 0.644 (95% CI 0.569–0.719) with a sensitivity of 88% and specificity of 35%. In the ROC analysis performed to evaluate the predictive power of the 27-R-HC level for autism, when the cut-off value was taken as 36.59 µg/L, the area under the curve was 0.639 (95% CI 0.563–0.715), the sensitivity was found to be 67%, and the specificity was 59% (see Figure 1).

### 3.5. Genotype and Allele Frequencies of LXRB Gene SNPs

**Figure 2:**
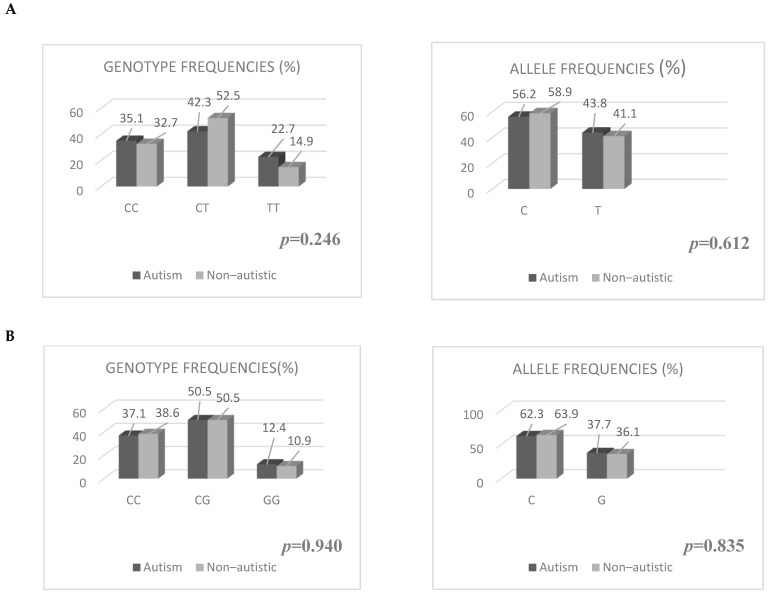
Comparison of Genotype and Allele Frequencies for rs2695121 and rs17373080 in the ASD and Non–autistic groups. (**A**): Comparison of Genotype and Allele Frequencies for rs2695121. (**B**): Comparison of Genotype and Allele Frequencies for rs17373080.

When genotype and allele frequencies were calculated for rs2695121 and rs17373080 in both groups, Hardy–Weinberg equilibrium was achieved. There was no statistically significant difference between the groups in terms of genotype and allele frequencies for rs2695121 and rs17373080 (see Figure 2).

## 4. Discussion

Our study assessed both central and peripheral parameters of cholesterol metabolism, which play an essential role in neuronal development in the ASD and non–autistic groups. The study sample comprised 210 children, 107 with ASD, and 103 non–autistic children matched in terms of age. In the study, the number of male children in the ASD group, where eligible children from the clinical sample were included, was greater than that in the non–autistic group. It is well known that male gender predominance is more conspicuous in clinical pictures of autism that are not accompanied by comorbid conditions [31]. Our study included children without an additional neurological or psychiatric diagnosis and who did not use medication to eliminate confounding factors that may arise in assessing parameters. Therefore, it was observed that the patients who were eligible were predominantly male. All statistical analyzes were performed considering the effect of gender on the results.

There are few studies in the literature comparing lipid profile levels in children with ASD with those in healthy children. Some of these studies have reported findings comparable to ours, demonstrating that serum cholesterol levels in children with ASD are elevated [32], whereas others demonstrate that serum cholesterol levels are lowered [33] or show no significant difference [34]. The first factor to consider when evaluating lipid profiles in children with ASD is gender. Although both genders were included in our study, the number of male children in the ASD group was higher than that in the non–autistic group, but the analyzes were conducted considering the gender effect. Gender had no effect on the lipid panel levels in either group, and there was a difference between the groups for total cholesterol, triglyceride, and LDL levels for both genders. In a study evaluating 25 men diagnosed with Fragile X, the genetic syndrome most associated with ASD, and 26 healthy men, a negative correlation was found between hypocholesterolemia detected in the patient group and ABC scale scores. Researchers have pointed out that hypocholesterolemia may be related to the pathogenesis of ASD [35]. Likewise, in another study of 29 men with ASD and 29 healthy men, the triglyceride levels were found to be significantly higher and HDL levels were found to be lower in the ASD group than in the non–autistic group [36]. Researchers have drawn attention to the presence of dyslipidemia in males with ASD. Although ASD is a disorder known to have a male gender predominance, evaluating cholesterol metabolism disturbances expected to be observed in ASD in both genders combined would highlight the effect of gender, which is a significant confounding factor on the data [37]. This will also allow researchers to link the findings to the etiopathogenesis of the disease regardless of gender.

Another important factor that differs between studies in the literature is the age range of the individuals evaluated. Our study included participants aged between 2 and 18 years. Lipid levels may vary depending on genetic and environmental factors throughout childhood. The total cholesterol level, which begins to increase at birth and stabilizes around the age of two, increases again before puberty and decreases slowly throughout puberty [38]. Aside from expected changes in the developmental process and genetic factors, environmental risk factors that may impact lipid panel profiles for children in the age range included in our study are limited. However, most studies investigating lipid levels in ASD have been conducted in the adult population [39]. It is known that environmental factors that may affect the lipid panel in adulthood are more common than those in childhood [5].

According to our findings, there was no difference in cholesterol levels between children in the ASD group who followed a restricted diet and those who did not. Similar to our study, 57 boys with ASD and 36 non–autistic boys with different diets were compared regarding lipid panels following a 3-year follow-up period in another study evaluating cholesterol metabolism in ASD. As a result, regardless of the diet, total cholesterol, non-HDL cholesterol, and total cholesterol/HDL values were significantly higher, and the HDL value was lower in the ASD group than in the non–autistic group. Researchers have suggested that it is essential to periodically screen for cholesterol disturbances in children diagnosed with ASD and to recommend individualized diet programs when necessary [32]. Similarly, in another clinical study showing increased cholesterol levels in children with ASD, no statistical relationship was found between differences in children’s dietary intake and lipid profile levels [36]. These findings indicate the significance of the effect of non-nutritional factors on lipid panel levels and metabolism in ASD.

When the groups were evaluated regarding oxysterol levels, 27-R-HC and 27-S-HC levels were significantly lower in the ASD group than in the non–autistic group, regardless of gender. There was no difference between the groups regarding 25-HC, 24-HC, and 24-S-HC levels. In recent years, increasing findings have pointed to the relevance of neuronal development, synaptogenesis, and cholesterol metabolism disturbances in ASD [5], making oxysterols a vital research topic in ASD etiopathogenesis and disease processes. Furthermore, the lack of specific markers for early diagnosis encourages research into novel biomarkers that link cholesterol metabolism disturbances with neurodevelopmental disorders such as ASD [40]. However, to the best of our knowledge, there is only one study in the literature that investigates the association between oxysterol levels and ASD. In their study, Grayaa et al. compared 36 children with ASD with 38 healthy children, and evidence showed that ASD is associated with altered levels of circulating oxysterol. Researchers have proposed that 24-HC, in particular, is an independent risk factor for the disease and may be used as a diagnostic tool. Unlike our study, no significant disease-related difference was detected in 27-HC and other oxysterol levels [17]. There are essential differences between this study and ours, which are thought to impact the findings. Our study included children aged 2–18 years to examine the association between oxysterol levels and the age and developmental process. Grayaa et al. evaluated children between the ages of 3 and 7. It is known that oxysterol levels may vary with age [41]; therefore, this difference between the sample groups is believed to be significant. It is also known that oxysterols are present at very low levels in the blood [15]. For this reason, it is thought that detecting the expected changes in ASD may be related to the sample size. Finally, while 31.8% of the ASD group in our study was evaluated as having severe autism, in the study conducted by Grayaa et al., the rate of children with severe autism was much higher, at 75.6%. Because the role of oxysterol levels in the etiopathogenesis of ASD has not yet been fully elucidated, it is thought that this difference may have an impact on the findings.

Although the research findings varied, they all hint at a disruption in brain cholesterol homeostasis. This may contribute to functional changes in the brain through different pathways, such as cytotoxicity, oxidative stress, apoptosis, and synaptic dysfunction [14], which are known to play a role in the physiopathology of ASD [42]. It is also known that disruption of brain cholesterol homeostasis leads to disruption of membrane permeability and the integrity of lipid rafts, whose importance has been indicated in the pathogenesis of many neuropsychiatric diseases [43]. Lipid rafts, which are thought to be platforms where cellular signal transmission is achieved, have been shown to play a role in synaptic plasticity and contribute to neuropathology such as Alzheimer’s disease, Parkinson’s disease, and Huntington’s disease [44]. Additionally, there is a significant overlap between signaling molecules or neurobiological pathways thought to be involved in ASD and synaptic proteins associated with lipid rafts [45]. In addition, studies have supported the functional connection between lipid rafts and synaptic dysfunction in ASD. The conclusion is that the dysregulation of cholesterol, an essential component of lipid rafts, leads to synaptic dysfunction [44]. Synaptic dysfunction is known to be the common basis of ASD [46]. Therefore, it is thought that abnormal cholesterol metabolism may be involved in the pathophysiology of autism through the dysfunction of lipid rafts.

To date, the most researched oxysterols and those suggested to be associated with neurodegenerative processes are 24-HC and 27-HC [47]. Several studies on brain oxysterol levels have emphasized the significance of the balance between 27-HC and 24-HC for neuronal functioning [48]. Significantly increased 27-HC/24-HC ratios in some brain regions because of disruption of the brain cholesterol production and destruction/excretion cycle have been linked to neurodegenerative processes in some studies [19]. In line with this information, it may be suggested that the low peripheral levels of 27-R-HC and 27-S-HC in our study may be associated with an increase and accumulation of 27-HC passing into the brain. This might be a cause underlying the high-volume gray matter structure in ASD and this hypothesis needs to be corroborated by postmortem and cellular studies [49]. In addition, it has been demonstrated that 27-HC, which passes into the brain through the blood–brain barrier following peripheral production, reduces amyloid-b peptide production [50]. This inhibition also supports the anabolic state and amyloid precursor protein accumulation [51]. Previous studies on ASD have shown that increased levels of amyloid precursor protein contribute to intracranial neuronal increase, as well as neuronal size and density, and promote gray matter expansion [52].

In our study, the levels of 27-R-HC and 27-S-HC were significantly lower in the ASD group. In contrast, blood lipids, including total cholesterol, triglycerides, and LDL, were higher, suggesting that the findings may indicate a disturbance in oxysterol formation processes.

According to our findings, the levels of 25-HC, 24-HC, and 24-S-HC declined with age in both the ASD and non–autistic groups. During a healthy developmental process, 24-HC blood levels decrease with age [41]. In the non–autistic group, 27-R-HC and 27-S-HC levels decreased with age, similar to other oxysterols. Remarkably, no association between the levels of 27-R-HC and 27-S-HC and age was detected in the ASD group. Besides this, oxysterol levels in the ASD group were found to be unrelated to autism severity and the presence of cognitive developmental delay/mental disability. These findings may suggest that oxysterol level changes observed in the ASD group may be a biomarker associated with the presence of neurodevelopmental disorder alone, independent of developmental stage, disease severity and cognitive status. Our current knowledge of the regulatory role of oxysterols in neuronal developmental processes suggests that studies with larger samples should confirm this hypothesis.

Although our findings suggest that there is no relationship between the identified *LXRƁ* SNPs and ASD, the significant results acquired in animal studies to date indicate that large-scale studies in which more polymorphisms are evaluated are needed to elucidate the relationship between ASD and *LXRƁ*.

### Limitations of the Study

In our study, the groups were not gender matched. However, this was considered in all statistical analyzes Furthermore, the lipid levels of the parents were not assessed in our study. Performing this evaluation in subsequent studies may provide important data to exclude familial cholesterol disorders. Because a homogeneous patient group was selected in our study, our findings can be more easily associated with ASD. However, future studies with more heterogeneous and larger samples are needed to investigate the effects of other neuropsychiatric comorbidities on plasma cholesterol and oxysterol levels in patients with ASD and to explain the common mechanisms of neuropsychiatric disorders. Additionally, because our study was not a follow-up study, no relationship could be established between treatment response and prognosis and cholesterol and oxysterol levels. Another critical point that we think is important is that oxysterol levels in the ASD group were found to be unrelated to autism severity and the presence of cognitive developmental delay/mental disability. However, this finding alone is insufficient for us to draw similar conclusions regarding other molecular pathways and possible biomarkers related to cholesterol and oxysterol. Population-based, generalizable further studies with comparisons to different markers are required on this topic.

## 5. Conclusions

Our study findings revealed that while total cholesterol, LDL and triglyceride levels were significantly higher in children with ASD compared to healthy developing children, 27-Hydroxycholesterol levels were significantly lower, and 27-Hydroxycholesterol has a potential role as a diagnostic marker for ASD. Although our research adds important data to the literature, it is thought that further community-based case-control studies should be conducted with larger samples to confirm and generalize the results. In addition, it remains an important research topic to determine whether the changes in cholesterol metabolism detected in ASD are related to possible mechanisms suggested at the cellular level, such as membrane lipid abnormalities, inflammation, abnormal immune activation, and oxidative stress. Moreover, molecular studies on brain oxysterol metabolism will expand our knowledge in this area. We hope that our study assessing peripheral and central cholesterol metabolism parameters in ASD will pave the way for future studies that will elucidate the association between autism and cholesterol metabolism disturbances and provide crucial data.

## Figures and Tables

**Table 1:** Sociodemographic characteristics of the group.

Sociodemographic Variables	ASD (*n* = 107) Mean (SD) *n* (%)	NA (*n* = 103) Mean (SD) *n* (%)	*p*
Child age (years) ^a^	9.54 (4.81)	10.18 (4.91)	0.301
Gender ᵇ			<0.001
Female	24 (22.4)	48 (46.6)	
Male	83 (77.6)	55 (53.4)	
Body mass index ^a^	19.28 (2.87)	18.98 (2.68)	0.463
Family type ᵇ			0.335
Nuclear	90 (84.1)	88 (85.4)	
Wide	9 (8.4)	6 (5.8)	
Divorced	8 (7.5)	7 (6.8)	
Parental Death	0	2 (1.9)	
Mothers’ age (years) ^a^	35.9 (5.18)	37.2 (5.18)	0.089
Fathers’ age (years) ^a^	38.71 (5.54)	39.26 (6.48)	0.266
Mothers’ education level ᵇ			0.659
Less than high school	39 (36.5)	38 (36.9)	
High school	36 (33.6)	38 (36.9)	
College degree or higher	32 (29.9)	27 (26.2)	
Fathers’ education level ᵇ			0.946
Less than high school	25 (23.4)	23 (22.3)	
High school	35 (32.7)	33 (32)	
College degree or higher	47 (43.9)	47 (45.6)	

ASD, autism spectrum disorder; NA, Non-autistic; SD, standard deviation; ^a^: Mann–Whitney U test ᵇ: Chi-square test.

**Table 2:** Clinical characteristics and average scale scores of the ASD group.

Clinical Characteristics	Frequency (N)	Percentage (%)
Autism Level		
Low	20	18.7
Mid	53	49.5
Severe	34	31.8
Global Developmental Delay		
Yes	89	83.2
No	18	16.8
Alternative Treatment		
Yes	28	26.2
No	79	73.8
Restricted Nutrition		
Yes	11	10.3
No	96	89.7
SCALES	Mean ± Standard Deviation	
Childhood Autism Rating Scale (CARS)	39.48 ± 6.97	
Autism Behaviour Checklist (ABC)		
Sensory	9.67 ± 4.28	
Relating Behaviors	11.05 ± 5.03	
Body and object use	10.97 ± 5.12	
Language	10.13 ± 4.93	
Social and self-help	10.63 ± 5.70	
Total	52.46 ± 21.47	
Repetitive Behavior Scale-Revised (*RBS*-*R*-*TV*)	25.44 ± 17.91	

**Table 3:** Mean lipid levels of children in ASD and Non–autistic groups.

	ASD Mean ± SD (Median)	NA Mean ± SD (Median)	*p*
Total cholesterol (mg/dL)	168.57 ± 31.18 (169)	155.6 ± 29.39 (157)	0.003 ^a^
Triglycerides (mg/dL)	113.6 ± 77 (96)	85.17 ± 50.1 (71.8)	<0.001 ᵇ
LDL (mg/dL)	100.05 ± 25.27 (98.1)	89.98 ± 28.07 (90,2)	0.008 ^a^
HDL (mg/dL)	55.70 ± 14.23 (53.8)	58.2 ± 12.18 (58)	0.179 ^a^

ASD, autism spectrum disorder; NA, Non-autistic; SD, standard deviation; ^a^: Student’s *t*-test; ᵇ: Mann–Whitney U test.

**Table 4:** Mean Oxysterol Levels of Children in the ASD and Non–autistic Groups and Their Relationship with Age.

	ASD Mean ± SD (Median)		NA Mean ± SD (Median)		*p*
25 HC (µg/L)	55.21 ± 40.72 (44.29)		47.44 ± 42.21 (34.76)		0.060 ^a^
Age ᵇ	rho	*p*	rho	*p*	
	−0.516	<0.001	−0.617	<0.001	
24S HC (µg/L)	56.27 ± 36.18 (48.08)		52.48 ± 44.83 (39.55)		0.098 ^a^
Age ᵇ	rho	*p*	rho	*p*	
	−0.504	<0.001	−0.522	<0.001	
24 HC (µg/L)	63.03 ± 44.34 (55.02)		64.60 ± 69.50 (43.84)		0.261 ^a^
Age ᵇ	rho	*p*	rho	*p*	
	−0.521	<0.001	−0.387	<0.001	
27R HC (µg/L)	34.73 ± 24.24 (24.24)		53.61 ± 41.68 (44.76)		0.001 ^a^
Age ᵇ	rho	*p*	rho	*p*	
	−0.007	0.944	−0.390	<0.001	
27S HC (µg/L)	34.63 ± 22.36 (27.43)		53.78 ± 39.86 (42.54)		<0.001 ^a^
Age ᵇ	rho	*p*	rho	*p*	
	0.039	0.695	−0.306	0.002	

^a^: Mann–Whitney U Test; ᵇ: Spearman correlation analysis; ASD, autism spectrum disorder; NA, Non-autistic.

## Data Availability

The datasets used and/or analysed during the current study are available from the corresponding author on reasonable request. The data are not publicly available due to restrictions e.g., privacy or ethical.

## Appendix A

**Table A1:** Primer pairs, amplicon lengths, and restriction digestion products for each SNP analysis.

SNP	Primer Pairs	Amplicon Lengths (bp)	Restriction Digestion Products (bp)
rs2695121	forward: GCTTAAGACCAAGCTGAAGGC reverse: CGGAGCAGCCTGTAGAATACA	225	C allele: 114 + 33 + 78 T allele: 147 + 78
rs17373080	forward: AGAAGCGCATGAATGAGCTAAATCCA reverse: GTAGATCTACTGGGACTTGGC	108	G allele: 84 + 24 C allele: 108
